# Persistence and Changing Distribution of Leishmaniases in Kenya Require a Paradigm Shift

**DOI:** 10.1155/2021/9989581

**Published:** 2021-10-18

**Authors:** Francan F. Ouma, Chrispinus S. Mulambalah

**Affiliations:** ^1^Masinde Muliro University of Science & Technology, Department of Biological Sciences, P. O. Box 190 50100 Kakamega, Kenya; ^2^Moi University, School of Medicine, Department of Medical Microbiology & Parasitology, P. O. Box 4606, Eldoret, Kenya

## Abstract

**Background:**

Leishmaniases present a major global public health problem, being responsible for between 40,000 and 50,000 deaths annually. The resultant morbidity affects the economic productivity and quality of life of individuals in endemic regions. As zoonotic disease(s), leishmaniases have become persistent with intermittent transmission and a tendency to disappear and reemerge, straining the fragile healthcare infrastructure in Kenya. There is a need to better understand disease(s) dynamics in Kenya. *Objectives of the study*. The status of leishmaniases in Kenya was reviewed to refocus and influence the attention of the scientific community and intervention strategies/policies on this persistent public health problem. *Methodology*. Electronic and manual literature were searched for relevant scholarly peer-reviewed published articles on leishmaniases. Literatures were obtained from PubMed, Medline, EBSCO, Host, ScienceDirect, and Google Scholar. *Findings*. The diseases are reported to be persistent as emerging and reemerging within and outside traditional endemic regions. Cutaneous leishmaniasis (CL) has maintained restricted foci in Nyandarua, Baringo, Nakuru counties, and Mount Elgon area in Bungoma County. Visceral leishmaniasis (VL) was most prevalent with cases in Baringo, Turkana, West Pokot, Isiolo, Kitui, Meru, Machakos, Marsabit, and Wajir counties. New VL cases/foci reported in formerly nonendemic regions/beyond traditional foci of Garissa and Mandera counties. Diagnostics, management, and control strategies have remained unchanged even in the face of changing disease epidemiology.

**Conclusion:**

Leishmaniases are emerging and reemerging persistent infections in remote rural settings in Kenya. The adopted intervention strategies have not been effective over the years, and this has led to disease spread to formerly nonendemic areas of Kenya. The diseases spread have been further enhanced by population growth and movement, environmental and climate changes, and social conflicts. It is evident that without a paradigm shift in control methods, diagnostic techniques, and treatment protocols, the diseases may spread to even more areas in the country.

## 1. Introduction

Leishmaniases are zoonotic diseases caused by obligate intracellular protozoan parasites of the family Trypanosomatidae in the genus *Leishmania*. Four species in the genus are commonly associated with human and animal diseases. These are *L. donovani, L. tropica, L. major,* and *L. aethiopica*.*Leishmania spp.* are normally transmitted through a bite of an infected Phlebotomine sand fly. In humans, *Leishmania spp.* are capable of causing a wide range of clinically distinct diseases. The three notable forms are visceral leishmaniasis (VL) caused by *L. donovani* and the most dangerous; cutaneous leishmanniasis (CL) caused by *L. tropica,* the most prevalent; and mucocutaneous leishmaniasis (MCL) caused by *L. donovani* and *L. major* cumulatively. The diseases are curable but cause high morbidity and death in humans as a result of low index of suspicion by health care professionals, late and or inappropriate diagnosis, and poor case management. If left untreated, VL has a high mortality rate of over 95% [1]. Global estimates of leishamaniases stand at about 12 million cases, with an annual incidence of between 0.7 and 1 M people and annual mortality rate of between 40,000 and 50,000 people. The Indian subcontinent holds about 80% of the world's leishmaniases cases. Other high endemic countries are Nepal and Bangladesh [1].

The East African region has the world's second most reported human VL cases after the subcontinent India between 29,400 and 56,700 cases annually. The general prevalence of VL in East African region is reported to follow this trend from the highest: Sudan, South Sudan, Ethiopia, Eritrea, Somalia, Kenya, and Uganda ([1, 2]; [1, 3–6]). Kenya has confirmed human cases for both VL and CL ([6], MOHK). It is possible that with population growth and movement, environmental and climate changes, and urbanization, leishmaniases distribution, spread, and prevalence patterns in Kenya have changed over time within and outside the usual foci.

The objective of the study was to review the status of leishmaniases in Kenya with specific reference to distribution within and outside known endemic foci and evaluate diagnostic, management, and intervention strategies. The purpose of the study was to refocus and influence the attention of the scientific community and disease intervention policy on leishmaniases in Kenya.

## 2. Sources of Information

Literature reviewed was obtained electronically and manually from various relevant sources. Electronic searches were carried out in PubMed, Medline, ScienceDirect, and Google Scholar. Reliable medical websites—national Institute of health (NIH), WHO, and the online research platform—EBSCO were used as sources of data and information. Ministry of Health Kenya (MOHK) publications on leishmaniases surveillance and response reports were accessed, and relevant information was extracted.

General information on leishmaniases was obtained by searching using the keyword—Leishman^∗^.

Literatures on the distribution of leishmaniases in Africa were obtained by searching (Leishmania^∗^) AND Africa. Literature information limited to Kenya was obtained by searching (Leishmania^∗^) AND Kenya.

## 3. Results

### 3.1. Historical Aspects of Leishmaniases in Kenya

The annual estimate of human leishmaniases cases in Kenya stands at about 4000 while 5 million people being are at risk of infection [7]. In Kenya, both VL and CL forms of the diseases have been reported. The first cases of VL in the country were reported from Wajir and Mandera counties in 1935, while the first CL case was described in 1969 [8]. Since then, the two diseases have generally persisted in two geographical areas: VL in arid, low-lying areas of some counties located in Rift Valley, Eastern, and North regions, whereas CL in semiarid lowlands to high plateaus in the Eastern, Rift Valley, Central and Western regions [9]. From these traditional areas, the diseases spread to other areas suitable for the survival of animal reservoirs and vectors and their interaction with man. The interaction of man vectors and leishmanial parasites led to the establishment of disease outbreaks within and outside the traditional foci [10].

### 3.2. Leishmaniases Endemic Counties of Kenya

Leishmaniases have been known to be endemic in parts of Kenya from as far back as the early 20th century. The earliest endemic foci identified in Kenya were in present-day counties of Turkana, Baringo, Kitui, Machakos, Meru, West Pokot, and Elgeyo Marakwet [11]. In these foci, the most prevalent form of disease was VL caused by *L. donovani* predominantly transmitted by *Phlebotomus martini*; however, cases of *Ph. orientalis* have also been reported [12]. Further studies confirmed that indeed Kenya was endemic for both VL and CL. This was found to be true when Baringo County was confirmed to be foci for both VL and CL during major outbreaks in the county reported in 2009 (Githure et al., 1986; [10, 11, 13, 14]). Since then, Baringo County is believed to have been the epicenter for the spread of VL and CL to neighboring/other counties with the involvement of various *Leishmania spp.* and vector species. For a long time, CL disease outbreaks in Baringo have been caused by *L. major*; *L. tropica* has been associated with the same disease in Laikipia, Samburu, Nakuru, Isiolo, and Nyandarua counties; *L. aethiopica* cases have been reported to cause CL in Mt. Elgon areas. Ph*. dubosci* is the vector for *L. major*, and Ph*. guggisbergi* is the vector for *L. tropica*. Vectors for *L. aetheopica* are *P. pediffer*, *P. longipes*, and *P. elgonensis* [11, 15]. Marsabit County, in Northern Kenya, with a population of 290,000 has become a major VL outbreak area of WHO concern (WHO 2017).

### 3.3. Leishmaniases Presentation and Morbidity Trend

**Figure 1:**
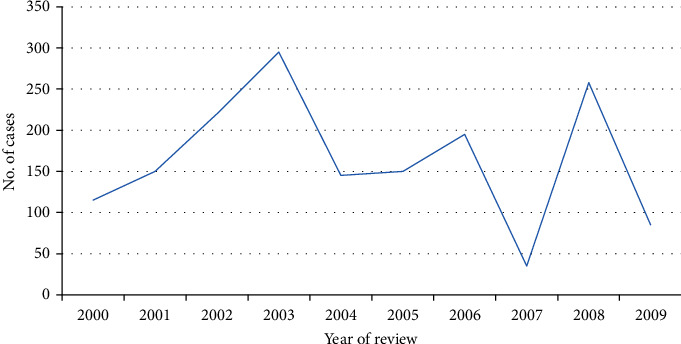
The visceral leishmaniasis trend in Kenya between 2000 and 2009 (Source: WHO, 2009).

In Kenya, low index of suspicion of leishmaniases by health care workers, late diagnosis, and poor case management of the disease results in high morbidity and mortality. If left untreated, VL has a high mortality rate (of about 95%). CL infections occur in simple or diffuse forms, whereas VL is characterized by hepatomegaly, splenomegaly, prolonged fever, anorexia, and weight loss [14, 16]. VL and HIV coinfection cases have been recorded in Kenya, and the association of the two is seen to hasten full-blown AIDS stage among HIV patients since both VL and HIV attack the defense system of the body, therefore triggering immuno supression. Cases of post-Kal-azar dermal leishmaniasis (PKDL) have been recorded among patients a few months after treatment with a notable prevalence ranging between 2 and 5% in treated individuals. PKDL patients are known to act as reservoirs of VL and therefore, PKDL case identification and treatment are important towards VL infection control (MOH). Reported cases of VL in Kenya for the period 2000 to 2009 are indicated in Figure 1.

**Figure 2:**
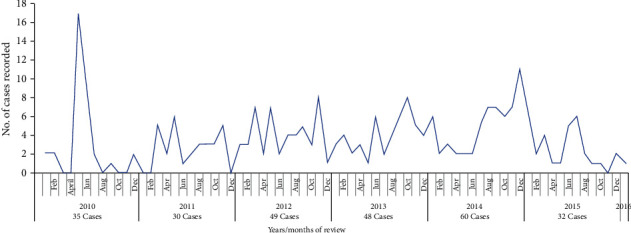
Cases of cutaneous leishmaniasis reported in Kenya from 2000 to 2016 (Source: [17]).

Despite little information available about CL in Kenya, the available literature indicates recurrent outbreak of the disease in the country over the years (Figure 2).

### 3.4. Emerging Leishmaniases Foci

**Figure 3:**
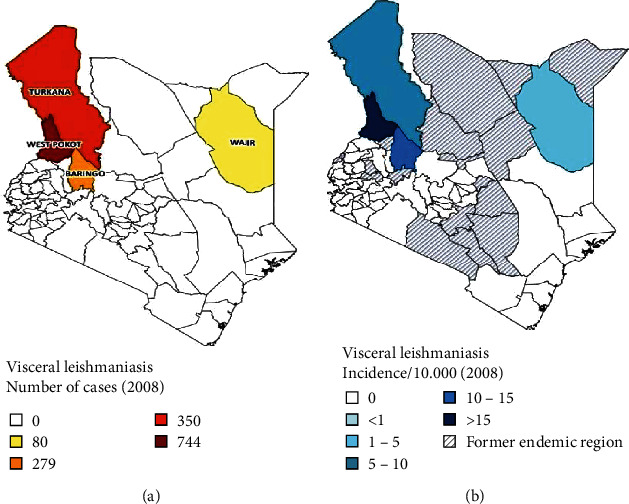
(a) VL cases in Kenya in 2008. (b) Areas reporting new cases of VL in Kenya by 2008 (Source: WHO Kenya Basic Country Data Total Population https://www.who.int/leishmaniasis/resources/KENYA.pdf?ua=1).

The distribution of leishmaniases in Kenya has been changing over the years due to several factors including social conflicts, population displacement, and effects of droughts in areas within and outside endemic regions including the Rift Valley and Eastern Region. In the north eastern part of Kenya, there are reports of increasing leishmaniases spreading to formerly nonendemic areas within Wajir and Mandera counties [10, 18]. The situation of leishmaniases in these regions may not be appropriately described due to the inaccessibility of the areas and diagnostic related challenges. Many of the emerging leishmanises foci are reported in northern and north eastern Kenya (Figure 3). The possible explanation is that there is a likelihood of imported cases from neighboring countries, South Sudan to the north and Somalia to the north east of Kenya. The movement of refugees from these counties into camps located in Kakuma, Garissa, Wajir, and Mandera could fuel the disease spread in this part of Kenya ([11]; MOHK 2018).

Furthermore, the emerging and reemerging leishmaniases in other regions may be associated with lack of knowledge and awareness of the diseases. Community involvement in outdoor activities within and or near endemic areas is more at risk of infection in both rural and urban settings. These together with increased cases of conflict related to cattle rustling enhance spread beyond known endemics (GItari et al.; 2018; MoHK 2018). The distribution of cases and incidence of VL in Kenya is presented in Figure 3.

VL infections in Kenya have recently been reported in Turkana, Baringo, West Pokot, Kitui, Machakos Marsabit, Tharaka Nithi, and Elgeyo Marakwet counties of Kenya [19]. On the other hand, the eastern slopes of Mount Elgon, the Abadere area, Naivasha, Laikipia plateau, and drier parts of Baringo form important emerging foci for CL in the country [10].

### 3.5. Diagnostic Approaches and Challenges

Microscopic examination of stained specimens of spleen aspirates to demonstrate the amastigote stage of the parasite has been the key VL diagnostic method used in Kenya (Wambugu et al., 2018). However, this method is highly invasive and sometimes results in fatal hemorrhage [14]. Alternative leishmaniases diagnostic tests include serological tests, for instance, enzyme-linked immunosorbent assays (ELISA) that detect leishmanial antibodies. ELISA technique is widely used in clinical routines and epidemiological surveys. Other serological tests like direct agglutination test (DAT) and immunochromatographic rapid dagnostic test (IRDT) are applicable but have varying sensitivity and specificity challenges [14].

To overcome sensitivity challenges, the molecular-based diagnostic method such as polymerase chain reaction (PCR) (and its variants) has become widely applicable. PCR-related techniques are reliable with high sensitivity and specificity but reagents are expensive for resource poor developing countries like Kenya.

Due to poor funding and lack of laboratory equipment and personnel, the diagnosis of leishmaniases in endemic areas in Kenya is based on disease, clinical presentation/history, and basic microscopic examination (MOHK). Therefore, there is a need for Ministry of Health to adopt innovative diagnostic tools with high sensitivity, specificity, reliability, feasible, and cheap.

### 3.6. Treatment Approaches and Challenges

Treatment of leishmaniases involves the use of drugs developed over half a century ago. In Kenya, sodium stibogluconate (SSG) still is the preferred treatment for VL and CL. SSG is toxic, and some patients die due to adverse effects, relapses, and unresponsiveness to treatment that has been reported in some patients in Kenya (Gitari et al., 2018; [20]).

Amphotericin B is widely used in Kenya to treat VL even though it is toxic and expensive, limiting its accessibility [21, 22].

Paramomycin has demonstrated good efficacy but is not widely used in Africa [21]. The most recent antileishmanial compound, miltefosine, has a high efficacy but highly susceptible to resistance and is teratogenic, hence limiting its use in expectant women patients [23].

In the absence of new, safe, affordable, and easily available drug(s), leishmaniases treatment and management will continue to be a challenge in Kenya.

### 3.7. Control and Prevention Approaches and Challenges

Control measures directed to the vector are widely used in Kenya. These include the use of insecticide impregnated bed nets, indoor residual spraying, and personal protection equipment (PPE) such as long sleeved shirts and boots among people in endemic areas, health education, and community awareness mobilization (MOHK). Despite these efforts, the rural nature of the diseases foci, the periodic drought and famine, and high levels of poverty and poor communication make it difficult to execute control and prevention of leishmaniases in Kenya. Moreover, control and prevention measures towards alternative hosts/reservoirs of VL are difficult to implement, especially where human reservoirs are involved, for instance, for PKDL cases which play a crucial role in the spread of VL. In the absence of a known reservoir, VL is considered an anthropogenic disease in Kenya (MOHK 2018). Human encroachment/economic activities within vector sand fly breeding areas and caves in endemic areas have continued to promote infections in new foci in Kenya even in the presence of intervention strategies [24].

There is scanty information on zoonotic transmission of leishmaniases in Kenya; although, hyrax and giant rat (*Cricetomys sp.*) are reported to act as reservoir hosts for *L. eathiopica* causing CL in Kenya [16, 25]. Other possible reservoirs of the disease(s) in Kenya are listed in Table 1.

Source: WHO (https://www.who.int/leishmaniasis/resources/KENYA.pdf?ua=1).

Even though the above recommended interventions have been applied and coordinated by WHO in Kenya, there is still evidence of an overwhelming rate of diseases transmission within and outside endemic foci. Therefore, there is an urgent need for a paradigm shift in the control and prevention of leishmaniases in Kenya.

### 3.8. Conclusions and Recommendations

Leishmaniases occur as emerging and reemerging infections within and outside endemic foci in Kenya. Most of the cases are reported in remote inaccessible, arid, and semiarid areas. The main intervention strategies have not been effective over the years, and this has led to disease spread to formerly nonendemic areas of Kenya. The disease(s) spread has been further enhanced by population growth and movement, environmental and climate changes, and social conflicts. It is evident that without a paradigm shift in control methods, diagnostic techniques, and treatment protocols, the diseases may spread to even more areas in the country.

To achieve effective control and management of the disease, the country needs to invest in the latest test kits for prompt diagnosis; procure and avail adequate drugs for the treatment of confirmed cases, engage skilled working force, enhance active and passive diseases and vector surveillances within and outside endemic areas, and offer continuous public health education to enhance health literacy on leishmaniases. A national and comprehensive leishmaniases screening program is recommended to map out old and new foci for effective diseases treatment and control.

Although scanty evidence pointing zoonotic transmission is available, elaborate studies should be done to identify animal reservoirs which may also extend the transmission beyond the formerly identified endemic areas.

Vector control approaches such as the use of impregnated bed nets, indoor residual sprays, and use of personal protective equipment should be recommended in the diseases endemic areas.

## Figures and Tables

**Table 1:** Reservoirs for *Leishmania spp.* parasites in Kenya (Source: WHO 2018).

Leishmania species	Clinical form	Vector species	Reservoirs
*L. tropica*	ZCL	*P. Guggisbergi*	*Procavia capensis*
*L. aethiopica*	ZCL, DCL	*P. Pedifer*, *P. aculeatus*	*Procavia capensis*, *Dendohyrax arboreous*, *Crycetomis sp*.
*L. major*	ZCL	*P. duboscqi*	*Tatera sp*., *Aethomys sp.*, *Arvicanthis* sp., *Meriones sp*.
*L. donovani*	VL, PKDL	*P. martini*, *P. celiae*, *P. vansomerenae*	
